# Debt-related regret and well-being in people resolving problem debts

**DOI:** 10.1186/s40359-025-03045-9

**Published:** 2025-07-01

**Authors:** Aidan Feeney, Simon McNair, Nicole Andelic

**Affiliations:** 1 School of Psychology, Queen’s University Belfast, University Road, Belfast, BT7 1NN UK; 2 Cowry Consulting, London, UK; 3 University of Manchester, Manchester, UK; 4 University of Aberdeen, Aberdeen, UK

**Keywords:** Debt, Regret, Explanations, Mental health, Decision-making, Gender

## Abstract

**Background:**

Regret is an often painful emotion experienced upon the realisation that a different decision would have led to a better outcome. As regret related to poor real world financial decision making has been neglected, here we examine whether people with debt problems regret the financial decisions that led to those problems, whether their explanations for their debt problems are associated with what they regret, and relations between regret, explanations for indebtedness, and well-being.

**Methods:**

We measured the well-being of people resolving problem debts (*N* = 260) who also rated the extent to which each of a set of factors (e.g., spending beyond their means, bad financial decisions, health, employment etc.) contributed to their debt problems and described up to three regrets about the events leading up to their problems with debt.

**Results:**

Although participants most often regretted spending or financial decisions, fewer than half of their regrets concerned such decisions and even amongst those who attributed significance to their financial or spending decisions, a substantial minority did not express spending regrets. Poor well-being was common in the sample and was associated with regrets about uncontrollable events and perceptions that employment issues contributed to the debt problem. Regrets over time taken to seek debt advice were common, although delays in seeking advice predicted better well-being. Gender significantly affected debt size, time before seeking advice, explanations for the debt problem, and well-being.

**Conclusions:**

These results suggest that (a) regret for myopic financial decisions may fade with time, even when those decisions have profound consequences, (b) people explain their debt problems in a variety of ways only some of which relate to their financial decision making, and (c) debtor well-being is associated with particular explanations and regrets. The results have implications both for delivery of debt advice and the design of interventions to encourage people to seek debt advice.

**Supplementary Information:**

The online version contains supplementary material available at 10.1186/s40359-025-03045-9.

## Debt-related regret in people resolving problem debts

Regret is a painful emotion experienced upon the realisation that a better outcome was possible had one made a different decision [37, 74]. Thus, regret and decision making are inextricably linked. Because regret is experienced when our decision making goes awry, the anticipation of the emotion can affect the decisions that we make [74]. Indeed, some theories in behavioural economics suggest that we choose so as to minimise the regret that might arise should our decision turn out badly [8, 43]. While much of the literature on regret describes studies of regret and anticipated regret in laboratory tasks (see [74] for review), there is also a literature on real life regrets, where people are asked to describe their biggest regret over their entire lifetime or in the recent past. People tend to regret their failures to act more than their actions [25], and studies reveal that decisions made in different areas of life, including education, career, romance and parenting, can lead to regret (e.g., [19, 58]). There is considerable evidence that intense lifetime regrets of this kind can have adverse effects on well-being (for a recent review, see [60]).

Somewhat surprisingly, financial regrets are not especially frequent when people are asked about their greatest regrets (see [58],for an exception see [13]) and there appears to have been rather little work focussing on people’s real-life regrets related to their economic decisions. Because people’s quality of life and their life chances are often determined by their economic decision making there is likely to be ample scope for regret about those decisions when they turn out badly, and the potential for such regrets to negatively affect well-being. There is work on regret in laboratory-based decisions about investment and asset allocation (e.g., see [5, 42]) and on post purchase regret in consumers (e.g. [1, 17, 33, 68]) but much of this work is carried out from a marketing perspective and focuses on regret as a barrier to repeat purchases or brand satisfaction. There appears to be less emphasis on the factors that might lead to problematic levels of regret in financial decision makers. One exception is the small amount of qualitative work that has been carried out on regret related to impulsive (e.g., [6]) and compulsive shopping (e.g., [20]). That small literature leads to several conclusions: (1) people do not always express regret for impulsive purchases,and (2) when they do express regret, consumers regret the money they have spent rather than the item they have purchased.

In contrast with the previous focus on regret associated with individual financial decisions, in this paper we will study the regrets of people who, as a result of bad financial outcomes, are engaged in a process of resolving their problematic debts. Debt becomes problematic when the debtor is unable to service their debt and many countries have legal processes to allow people to resolve their problem debts. For example, an Individual Voluntary Arrangement (IVA; see [65]) in the UK allows the debtor to negotiate with their creditors so that a fixed amount may be repaid monthly for a fixed period (usually five years). Although creditors typically receive only a fraction of what they are owed, the debt is entirely written off, as it might be under bankruptcy. Similar arrangements are available in other countries (e.g., Chapter 13 bankruptcy in the US and Personal Insolvency Arrangements in Ireland).

Indebtedness is an increasingly significant social problem. For example, in November 2023 the average credit card debt per UK household had risen by 8.6% to £2452 over the course of the year [67]. There were 25% more requests for registered breathing spaces, in which the debtor is given 60 days, in 2023 than in 2022 [66]. These trends are worrying because indebtedness is associated with low levels of psychological well-being generally [11] as well as with anxiety and depression more specifically (for a meta-analysis, see [56]). Studies of pathways into indebtedness have also revealed that debt can occur for very different reasons. For example, Webley & Nyhus [69] contrast dispositional routes into debt with life cycle routes. Thus, people can become indebted for reasons to do with self-control: poor self-control may lead to impulsive financial decision making which subsequently necessitates debt. Alternatively, people may take on debt for rational reasons due to life events when their income exceeds what they must spend on necessities (see [52]). Because studies of people with problematic levels of debt are relatively rare (although see [49, 53]), we do not fully understand how, in a minority of cases, indebtedness becomes a problem (but see [31]). However, a wide variety of dispositional, attitudinal and situational factors seem to play causal roles (see, e.g., [63]). Certainly, we would expect a proportion of debt due to dispositional factors to become problematic as psychological factors, such as impulsiveness or present bias, which cause some people to become indebted in the first place are also likely to interfere with their ability to pay off those debts [38]. Debt taken on in the expectation of short- or medium-term increases in income can also become problematic when, due to external events (problems with the economy, relationship problems, or health problems) the debtor is no longer able to service their debt. For example, in the US and China, medical debt is a widespread problem [41, 72] and relationship breakdown, job loss and income shocks are all associated with financial problems [45].

Whereas much previous work has focussed on the causes of indebtedness or on its psychological and economic consequences (see [38]), to the best of our knowledge, there has been no exploration of regrets related to debt and their effects on well-being. Studying the regrets of people with problematic levels of debt has the potential to shed light on a number of issues. First, if, as is claimed, there are different causal factors in play, then we might expect to see different patterns in people’s regrets depending on how they explain those debts. For example, debtors who attribute their indebtedness to their own financial decisions and spending may regret spending-related decisions more than debtors who do not. On the other hand, debtors who attribute their debt problems to situational factors may express regrets for events outside their control.

An analysis of debt-related regrets might also increase understanding of how what we regret changes over time [25], [36]. One possibility is that, amongst debtors who attribute their indebtedness to their own financial decision making, debt-related regrets may be almost entirely focussed on those myopic spending decisions. Under a functional account of counterfactual thinking and regret [50, 57], such regrets help us to make better choices next time. Alternatively, ‘hot’ regrets [26] for bad financial decisions may cool over time and be replaced by regrets related to other aspects of the debtor’s situation. Relatedly, it has been shown that, over time, people come to regret missed opportunities more than they do failures of self-control [36]. Similarly, people may come to regret their bad financial decisions less as time passes even if those decisions were central to their subsequent debt problems.

Problem debt is known to be associated with poor mental health (see [56]) and, under certain circumstances, regret is also associated with poor mental health. Thus, studying regret in people resolving problem debts has the potential to provide new insights into the determinants of poor well-being in debtors. One possibility is that number or intensity of debt-related regrets might predict poor well-being [35, 71]. Alternatively, the nature of people’s regrets may differentially predict well-being (see [39, 47]) with particular types of regrets more likely to be associated with poor mental health. Debtor well-being is an important issue for debt advisors and understanding the emotional correlates of poor well-being in people with problem debts has potential to inform and improve debt advice strategies.

An additional reason to study debt-related regrets is that doing so may provide insights into how to help people avoid such regrets by taking steps to avoid problematic levels of debt or to address such debts once they have occurred. Most obviously, if people do regret their spending decisions, then interventions designed to prime the anticipation of regret in the face of risky financial decisions (e.g., [62]) may be validated. Another possible outcome relates to persuading people to seek debt advice when their debt problems become clear. Debt advice is difficult to deliver [3] and mental health problems can impede people’s ability to adhere to the advice that they receive [2]. Moreover, in the UK there is large unmet need for debt advice [48]. Although much of this unmet need is likely due to demand for advice exceeding supply, anecdotal evidence suggests that some debtors do not seek advice when they need it. Accordingly, a particular focus of the current study will be how long participants report waiting before they sought debt advice, whether length of time waited is related to mental health or debt size, and whether debtors express regret for the length of time they waited before seeking advice.

A final reason for studying regrets related to debt advice is to examine whether those regrets differ by gender. Although recent research reveals an absence of gender differences in attitudes towards debt [9], there is evidence of gender differences in debt tolerance [24] and that amongst undergraduates, men borrow more than women [18]. On the other hand, older women appear to be more susceptible to debt problems than older men [55]. The evidence about gender differences in regret is equally mixed. For example, there is some evidence that women experience regret more intensely than men following failed hypothetical investment decisions [4], and that women experience more regret than men following unplanned purchases [14],although for evidence that men experience more regret following unplanned purchases, see [61]. The ‘repurchase effect’ in investment decision making [64] – where people are unwilling to repurchase a stock if its value rose after they sold it – is correlated with the degree of regret felt about the price rise [23]. This effect is larger in women, who experience more regret about the price rise than do men [40]. Because of this (albeit, somewhat mixed) evidence for gender differences in debt experiences and regret related to financial decision making, we included gender as a factor in our statistical analyses. Any gender differences may have implications for financial counselling of men and women with problem debts.

## The current study

The current study was part of a larger investigation of emotions and well-being in people resolving problem debts carried out in November and December 2012. Although collected in the wake of the post-2008 financial crisis, these data are particularly relevant now in the aftermath of the COVID-19 pandemic. Recent statistics [51] show post-pandemic household debt to income ratios in the UK (averaging 139% in the period 2020–2023) approaching levels when our data were collected (averaging 142% in the period 2012–2016). Moreover, recent evidence points to associations between income loss during the pandemic, ensuing financial problems, and deterioriation in mental health [10], and between mental ill health and measures of both subjective and objective hardship [34]. Our study was designed to examine relations between debtors’ debt-related regret, their perceptions of the causes of their debt, and their well-being. Thus, its results are relevant to understanding the experiences of people with post-pandemic financial problems and how best to advise them.

In our study, we asked a sample of people resolving their problem debts (the vast majority of whom were in an IVA) about the size of their debt, how long they waited before seeking debt advice, to explain their indebtedness by rating the extent to which a number of factors had contributed to their debt, and to describe three regrets about their financial difficulties. To measure the degree to which participants explained their indebtedness in terms of their own financial decision making we asked questions about living beyond their means and poor financial decision making. To capture explanations in terms of situational factors we asked about issues with employment, issues with relationships, failure of a business, and health problems. To address questions about associations between well-being and both regret and type of explanation, we asked participants to complete a measure of general well-being.

We used participants’ answers to these questions to address a number of research questions. First, we were interested in how debtors explain their pathways into indebtedness, i.e., their beliefs about the extent to which each of the contributory factors had played a role in their debt problems. As well as examining which explanations are offered most frequently, we were interested in whether people tend to focus on their own decision making versus situational factors or assign weight to both kinds of explanation.

Second, we sought to examine what debtors regret overall. That is, we were interested in the proportion of regrets that were about spending decisions, or other factors such as the consequences of those decisions, events beyond the debtor’s control, or decisions to delay seeking debt advice. A functional account of regret which explains the emotion as targeting aspects of decision making which need to be improved (see [57]) might predict that amongst people with problem debts, regrets over spending decisions will be more frequent than other types of regret. However, as earlier work suggests that what is regretted may change over time [25], [36], with the passing of time, debtors may come to regret other aspects of their situation. Thus, people with problem debt may not regret spending decisions as often as their financial situation suggests they might.

Third, we examined relations between people’s explanations for their indebtedness and the content of their regrets. Problem debts can be caused by bad financial decisions on behalf of the debtor or by life events over which they have little control. When problem debts are perceived to have been caused by uncontrollable life events, it is unlikely that debtors will regret their spending decisions. Thus, a stronger test of the hypothesis that people with problem debts will regret their financial decision making is to distinguish between those who explain their debts in terms of their financial decision making and those who don’t. The functional argument described above suggests that people who attribute their debt to their own financial decisions should tend to overwhelmingly regret those decisions. Accordingly, we analysed regret content conditional on the belief that financial or spending decisions had played a large role in the debt.

A fourth regret-related question concerned the distinction between regret for action and inaction. Although the standard pattern is that regret for inaction is more common than regret for action [25], it is unclear that this pattern will hold for debt-related regrets. In particular, many of the factors which cause action regrets to be regretted less (e.g., finding silver linings, action being more explicable in retrospect than inaction) may be less likely to operate when faced with a wholly negative outcome such as severe problem debts.

A fifth question concerned associations between well-being and factors such as debt size and time to seek debt advice as well as explanations for problem debts and regret content. One obvious hypothesis here is that poorer well-being will be found in people with larger debts. Another hypothesis is that people who attribute their indebtedness to their own decision making or who regret their own financial decisions or the time it took them to decide to seek advice will have poorer well-being because they make more internal causal attributions for their negative situation [54]. However, internal causal attributions may also be indicative of being relatively high in internal locus of control [59], which is known to be positively associated with mental health [12, 73]. Thus, an alternative possibility is that making external rather than internal causal attributions (attributing causal significance to job loss or health problems, for example) and regretting uncontrollable events may be associated with poor mental health. On the basis of previous work showing that intense regrets can be associated with poor mental health [60], we asked participants to rate the intensity of each of the regrets they described and planned to examine relations between regret intensity and mental health.

Finally, we were interested in the role played by debtor gender in explanations for and emotional responses to problem debt. Thus, we included gender as a factor in our analyses in order to test exploratory hypotheses about interactions between debtor gender and a) ratings of contributory factors and b) regret content.

## Method

*Participants* 260 (129 male) participants from a larger sample recruited from amongst the clients of a reputable debt resolution company local to the first author’s university were included in this study. Potential participants were contacted via invitiation email from an employee at the debt resolution company. The age of participants who accepted this invitation ranged from 29–78. Mean age was 47.59 years (s.d. 10.44). Fourteen percent of participants were single, 55% married, 23% separated or divorced and 8% described their marital status as ‘other’. Seventy-nine percent reported that they had children. Twenty-five percent of participants had been educated to GCSE level, 20% to A-level, 32% to university level and 22% described their educational level as ‘other’.

The study was approved by the School of Psychology Research Ethics Committee at Queen’s University Belfast. Given that the research was funded by the company of which participants were clients, and the sensitivity of the data which might have allowed individual participants to be identified by employees of the company, even after anonymisation, a condition of ethical approval of the study was that participant responses would not be shared beyond members of the research team which did not include company employees. Accordingly, as prior to their consent being obtained participants were informed that their responses would not be shared, we are not able to share our data due to ethical restrictions.

*Materials* Once they had provided their consent, participants completed an online questionnaire in four parts. The first part included seven questions about the participant, eight questions about their experience of debt advice and three questions designed to measure the extent to which participants were subject to present bias when thinking about money. We describe only the questions about the participants and the first question about their experience of debt advice here as none of the other questions were relevant to this study. All of the questions described here are reproduced in the supplementary materials.

In order, Questions 1–6 asked participants to indicate their age, to state their gender (male, female), to indicate their marital status (Single, Married, Separated/Divorced, Other), whether they had children, to what level they were educated (GCSE, A-Level, University, Other), and to indicate which formal debt resolution mechanism they had chosen (bankruptcy, Individual Voluntary Arrangement, Debt Management Plan, yet to decide). Question 7 asked participants to indicate the approximate size of their debt by choosing one of six responses (up to £20,000, between £20,001 and £40,000, between £40,001 and £60,000, between £60,001 and £80,000, between £80,001 and £100,000, more than £100,000). The only debt advice-related question relevant to the current study asked participants how many months it was after they realised that they had a serious debt problem before they sought help.

The second section of the questionnaire concerned shame proneness and will not be described here. The third section asked participants to indicate how they felt about their situation. First, participants were asked, on a five-point scale anchored Very little/No contribution and Very large contribution, to indicate the extent to which each of six factors had contributed to their debt. These factors were Living beyond means (e.g., expensive purchases); Poor financial decisions (e.g., bad investments, taking out a large mortgage); Failure of a business; Employment issues (e.g., redundancy, unemployment); Relationship issues (e.g., Separation or divorce, bereavement); Health problems (e.g., medical bills). We did not use an already existing measure for this part of the study but chose factors for inclusion on the basis of consultation with employees at the collaborating debt resolution company.

There followed a series of questions about the factors that had prompted participants to seek advice from the collaborating company and the quality of the advice they had received. Next, participants were asked how they would undo the event that led up to their financial difficulties. We will not consider these questions here.

The final questions in the third section of the questionnaire concerned regret. Participants were asked whether there were things about the run-up to their financial difficulties which they regretted. If there were, they were asked to describe up to three things about which they felt regret. Following each description, participants were prompted to rate the intensity of each regret on a scale from 1 (very little regret) to 5 (a lot of regret). Participants’ tended to report very intense levels of regret and, as there was little variance in regret ratings (82% of all regrets rated were assigned a score of 5), we will not present an analysis of those ratings here.

In the final section of the questionnaire, participants completed the GHQ12 [28], a widely-used 12-item measure of acute mental health which asks whether participants have recently been feeling and behaving in ways that are consistent with poor psychological health, e.g., having recently lost sleep over worry, and having recently been feeling depressed. The GHQ12 is a highly reliable measure and a recent meta-analysis of 20 studies found a mean coefficient alpa of 0.84 (individual alphas ranged from 0.7–0.93; [70]). Participants respond to each item on a four-point Likert scale ranging from 0 to 3. For screening purposes in clinical contexts responses are scored bimodally, so that those consistent with psychological health (0 and 1) are coded ‘0’ and those suggesting psychological ill-health (2 and 3) are coded ‘1’. This results in a score between 0 and 12 where higher scores indicate greater psychological ill-health. For research purposes, responses are coded on the 0–3 scale. We use both scoring methods here; the bimodal method for comparing our sample to other samples, and the Likert method for statistical analyses involving GHQ scores. The coefficient alpha score for this sample, scored using the Likert method was high, α = 0.96.

*Procedure* The email from the collaborating debt resolution company to a sub-section of their clients asked them to participate by clicking on a link which brought them to the online questionnaire. Participation was entirely anonymous. To thank them for taking part, once participants had completed the questionnaire, they were invited to participate in a lottery for one of four £50 vouchers.

*Analysis/Coding* Participants described a total of 601 regrets or 2.31 (s.d. = 0.86) regrets each. Regret descriptions were coded in two different ways. First, based on our research questions and careful initial reading of the regret descriptions, we derived a five category content coding scheme which included spending regrets (regret about spending money which could concern specific purchases or regret over spending habits); delay regrets (regret about not seeking help sooner); event regrets (regret about a particular event, outside the individual’s control); consequence regrets (regret related to consequences of the debt for the debtor or for those close to the debtor); and character regrets (regret related to the debtor’s emotional state or personal characteristics). The categories were not exclusive. That is, more than one code could be applied to the same description. This meant that five coding decisions were made about each description resulting in 3005 coding decisions. Two coders made these decisions independently. They agreed on 95.5% of decisions and disagreements were resolved by a third coder. 85.5% of regrets were coded into at least one of these categories.

Regret descriptions were also coded as concerning an action (describing a decision or behaviour that contributed to the debt problem), an inaction (something the individual did not do, or delayed doing that contributed to the debt problem), both action and inaction, or impossible to code. As these codes were mutually exclusive, each of two coders independently made 601 coding decisions. They agreed on 92.5% of decisions and disagreements were once again resolved by a third coder.

## Results

### Sample characteristics

Of the 260 participants in the sample, 247 were in an IVA, two had declared themselves bankrupt, five were in a debt management plan and six were in the process of deciding how to resolve their problem debt. The median amount owed was £40–60,000. Relevant to our question about whether there are gender effects related to debt, men (median debt £40,001-£60,000) reported owing significantly more than women (median debt £20,001-£40,000), U = 7286, *p* = 0.048. Relevant to our question about how long people waited before seeking debt advice, participants indicated that after realising that they had serious debt problems they waited an average of 14.6 months (s.d. = 13.37) before seeking help. Men (mean = 16.85, s.d. = 15.51) reported waiting longer than women (mean = 12.51, s.d. = 11.59), t(240.59) = 2.55, *p* = 0.01, d = 0.32.

When scored bimodally, the modal GHQ12 score of the 251 participants who completed the measure was 0 and scores ranged from 0 to 12. Although the modal score was 0, 48% of participants scored higher than the cut-off of 2 used to identify people likely to have mental health problems. This rate of above cut-off responding is considerably higher than rates previously reported in population level studies using the GHQ12. For example, Hoeymans et al. [30] reported that only 13% of their Dutch participants scored above the cut-off. Thus, there is likely to have been a much higher rate of mental health problems amongst the participants in this study than we would expect to find in the general population.

Well-being scores were associated with other demographic factors. For example, the mean Likert-scored GHQ12 score for women (mean = 15.46, s.d. = 10.31) in the study was significantly higher than for men (mean = 12.50, s.d. = 9.12), t(249) = 2.41, *p* = 0.02, d = 0.30. Although the zero-order correlation between GHQ12 scores and amount owed was non-significant, r = 0.03, *N* = 251, *p* = 0.59, greater time taken to seek debt advice was significantly associated with better well-being, r = −0.19, *N* = 250, *p* = 0.003. More time taken to seek debt advice was also associated with higher debts, *r* = 0.15, *N* = 259, *p* = 0.02. A multiple regression predicting well-being from amount owed, gender, and time taken to seek debt advice predicted significant amounts of variance in well-being, F(3, 246) = 5.01, *p* = 0.002, and revealed that both time taken to seek advice, β = −0.13, *p* = 0.005, and gender, β = 2.74, *p* = 0.03, independently predicted GHQ12 scores.

### Ratings of contributory factors

Participants rated the extent to which each of six factors contributed to their debt issues. Relevant to the research question about how debtors explain their indebtedness, examination of mean factor ratings in Table 1 suggests that living beyond their means was perceived as the factor that contributed the most to their debt issues, closely followed by bad financial decisions. Employment issues were also perceived to be an important factor. These means suggest that although spending decisions were perceived to be an important factor in how participants explained debts, many participants may have ended up in debt due to adverse life events such as unemployment, relationship breakdown or poor health. Strikingly, there are no strong positive or negative correlations between any of the factors with the strongest correlation occurring between the ‘living beyond my means’ and ‘bad financial decisions’ factors. At 0.24, this correlation would normally be viewed as weak. The absence of (a) strong positive correlations between the two financial factors or the three situational factors, and (b) strong negative correlations between financial and situational factors suggests that participants may have explained their pathway into indebtedness via a mixture of dispositional and situational factors.

**Table 1:** Inter-correlations between GHQ12 scores and factors contributing to debt problems Means (s.d.) and inter-correlations from GHQ12 scores and ratings of the extent to which each of six factors contributed to the debt problem

		Contributing Factor					
	Mean (s.d.)	Living beyond means	Financial decisions	Business failure	Employment	Relationship	Health
GHQ 12	13.95 (9.81)	-.09 *N* = 251	-.03 *N* = 251	0.10 *N* = 245	.14* *N* = 246	-.10 *N* = 247	.06 *N* = 251
Living beyond means	3.70 (1.43)		.24*** *N* = 256	-.16** *N* = 250	-.11 *N* = 252	.01 *N* = 252	-.17** *N* = 251
Financial decisions	3.61 (1.46)			.09 *N* = 251	-.02 *N* = 253	-.02 *N* = 252	-.07 *N* = 252
Business failure	2.06 (1.64)				.19** *N* = 248	-.09 *N* = 249	.12 *N* = 248
Employment	3.16 (1.73)					-.06 *N* = 250	.16* *N* = 249
Relationship	2.55 (1.74)						.08 *N* = 249
Health	2.05 (1.56)						

*Indicates *p* <.05

**Indicates *p* ≤.01

***Indicates *p* ≤.001

A 6 (Factor) × 2 (Gender) ANOVA on the data of 245 participants who provided ratings for all six factors revealed a main effect of Factor on ratings, F (4.57, 1110.04) = 57.14, *p* < 0.001, η_p^2 = 0.19. Bonferroni corrected t-tests comparing the means involved in this main effect revealed that each mean was significantly different from all other means with the exception of the mean ratings for bad financial decisions and living beyond my means and the mean ratings for failure of a business and health problems. Thus, financial factors were most strongly rated as having contributed to participants’ debt problems.

**Fig. 1:**
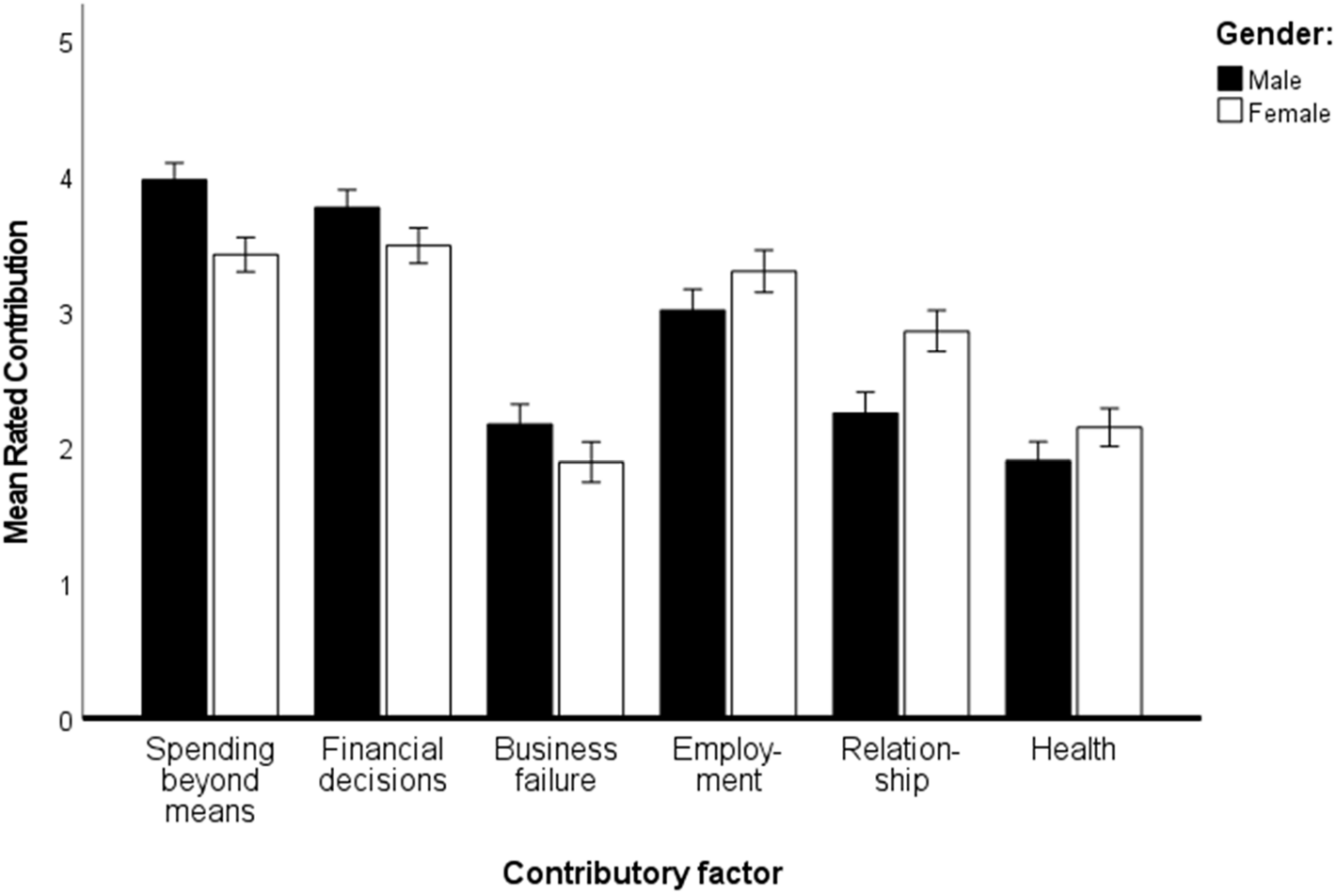
Mean ratings for contributory factors broken down by gender. Error bars represent standard errors

Relevant to the research question about effects of debtor gender on explanations for debt, gender interacted significantly with Factor, F(4.57, 1110.04) = 4.83, *p* < 0.001, η_p^2 = 0.02. The means involved in this interaction are displayed in Fig. 1. Bonferroni-corrected post hoc comparisons revealed only two significant differences between ratings due to gender: men rated spending beyond their means a stronger contributing factor than did women (*p* = 0.002), and women rated relationship problems as making a stronger contribution to their debt than did men (*p* = 0.006).

### Regrets

*Regret Content* Five independent coding decisions were made about each regret meaning that each regret could be coded as belonging to up to five content categories (spending, delay, events, consequences, character). The average number of content categories to which a regret was assigned was 1.23 (s.d. = 0.82). Out of the total 601 regrets, 87 (14.5%) were unassigned, 342 (56.9%) were assigned to just one category, 128 (21.3%) were assigned to two categories, and the remaining 44 (7.3%) were assigned to more than two categories. With the exception of delay regrets, where participants uniformly expressed regret for delay in seeking debt advice, the content of other regret types varied. For example, although many financial regrets concerned actual purchases, lack of self control or lack of planning, others referred to financial decisions made in an attempt to solve debt problems (e.g., borrowing additional money, gambling to pay back debts, payday loans, use of revolving credit, believing financial advisors). With respect to regrets categorised as being outside the participant’s control, many regrets referred to health or employment issues. For example, one participant regretted ignoring and then delaying treatment for a health problem while another regretted losing their job due to ill health. Consequence regrets concerned either consequences for the debtors themselves or for other people; several participants expressed regret for the consequences of their financial situation for their children (regret over depriving children of food, letting people down). Finally, character regrets consisted of regrets expressed about either the emotional consequences of their circumstances (shame, being humbled), or the personality traits that participants perceived to have contributed to their problems (being ambitious, not standing up to a spouse, hiding things from a partner, being disorganised when it came to money).

The average proportion of participants’ regrets in each of the five content categories are to be seen in Table 2 alongside the percentage of participants expressing at least one regret of each type. Notably, at 0.37, the proportion of spending regrets was less than we might have expected although 58% of participants described at least one regret related to spending. A Friedman ANOVA revealed a main effect of category on regret proportions, χ^2^(4) = 42.18, *p* < 0.001. Bonferroni adjusted post hoc Wilcoxon Signed Rank Tests revealed that the mean proportion of participants’ regrets that were of spending was significantly higher than all other mean proportions (all ps < = 0.01). None of the other mean proportions were significantly different from each other. Mann Whitney U tests carried out on each category separately revealed non-significant effects of gender on regret proportions.

**Table 2:** Regret prevalence broken down by spending score

	Regret Category				
	Spending	Delay	Events	Consequences	Character
Mean proportion (s.d.) when spending score < median	.31 (.37)	.23 (.35)	.25 (.35)	.26 (.34)	.24 (.30)
% participants describing ≥ 1 regret	50	40	42	44	46
Mean proportion (s.d.) for spending score > median	.42 (.38)	.15 (.25)	.14 (.25)	.21 (.30)	.25 (.32)
% participants describing ≥ 1 regret	64	32	30	40	48
Overall mean proportion (s.d.)	.37 (.38)	.19 (.30)	.20 (.31)	.23 (.32)	.25 (.31)
Overall % participants describing ≥ 1 regret	58	35	36	41	47

Mean proportion of regrets falling in each of five categories and number of participants expressing at least one regret in each category broken down by whether participants indicated that spending/financial decisions made a significant contribution to their debt problem. Proportions sum to more than 1 as individual regrets could be coded as belonging to more than one category

*Regret co-occurences *The results above suggest that regrets of spending, although not as universal as might have been expected under a functional account of regret (e.g., [57]) are somewhat dominant. However, despite the frequency with which participants report spending regrets, the pattern may not be straightforward to interpret. That is, given that just under 30% of the regrets described by participants received more than one coding, it is possible that spending regrets tended to systematically co-occur with other types of regret. For example, events outside the participants’ control might be regretted and they in turn might lead to regret of an earlier spending decision. Thus, one might regret having bought a new car because one subsequently lost one’s job, or because the payments on the car were unaffordable in the first place. To rule out the possibility that, for example, subsequent events systematically led participants to regret earlier spending decisions, we examined the co-occurrence of spending categorisations with each of the other four regret content categories. In total, 218 regrets were of spending. Seven (3.2%) of those regrets were also coded as expressing regret for a delay, only 32 (14.7%) as also expressing regret for events beyond the participant’s control, 35 (16.1%) as also expressing regret for consequences of the debt, and 42 (19.3%) as also expressing regret about aspects of the debtor’s character. It is striking that regrets about consequences of the debt were just as likely to co-occur with spending regrets as were events outside the debtor’s control. This pattern suggests that participants did not express regret for spending decisions only in certain circumstances such as when subsequent events outside their control impacted on their ability to service pre-existing debt.

*Debt explanations and regret content* Surprisingly, although all of the participants in this study were resolving problematic debts, only 37% of the regrets expressed concerned spending or other financial decisions. This appears problematic for a purely functional account of regret. However, a fairer test of the functional argument would be to examine regret content distributions dependent on whether people blame their spending or other financial decisions for their debt. Thus, we examined whether people who attribute their accrual of problem debt to overspending are particularly likely to regret their decisions to spend money. Prior to this analysis, we used the ‘spending beyond my means’ and ‘bad financial decisions’ items to compute a spending score and compared the regrets of participants (*N* = 124) whose score was below the median (8) to those whose score was at or above the median (*N* = 132). Of participants at or above the median on this combined spending scale, 64% reported at least one spending regret and the mean proportion of regrets that concerned spending in this group was 0.42. 50% of all participants in the other group reported at least one spending regret and the mean proportion of their regrets that were of spending was 0.31. Regret type proportions and number of participants reporting at least one regret of each type may be seen in Table 2, broken down by the perceived contribution of spending decisions to the debt. Separate Friedman ANOVAs on participants for whom spending beyond their means was or was not an important contributory factor revealed a significant effect of regret content for the former, but not the latter group. That is, for participants who scored below the median on the spending measure, the effect of regret content was not significant, χ^2^(4) = 4.27, *p* = 0.37. On the other hand, there was a significant effect of content for participants who scored at or above the mean on the spending measure, χ^2^(4) = 48.87, *p* < 0.001. Second, we used Mann Whitney U tests to compare the two groups on the mean proportion of regrets in each category. These revealed a bigger proportion of spending regrets described by participants who perceived spending decisions to have made a larger rather than smaller contribution to their debt, U = 6886, z = 2.30, *p* = 0.021, and a smaller proportion of regrets concerning events beyond their control, U = 6900.5, z = −2.54, *p* = 0.011.

Regrets due to action/inaction To answer the research question about whether participants’ debt-related regrets are for inaction rather than action, as might be expected from a reading of the regret literature (e.g., [25]), we categorised each regret in the full sample as concerning action, inaction, or both. Half (mean = 0.50, s.d. = 0.38) of participants’ descriptions concerned regret for action, and only one in three (mean = 0.34, s.d. = 0.37) concerned regret for inaction. The remaining descriptions could not be coded or mentioned both action and inaction. A Wilcoxon Signed Ranks Test revealed that the proportion of participants’ regrets that concerned action was significantly greater than the proportion that concerned inaction, z = 3.41, *p* < 0.001. Strikingly, the tendency for participants to attribute their regrets to action rather than inaction was entirely reversed for regrets about delays in seeking debt advice. There were 495 regrets which could be categorised as being for action or inaction of which 99 related to delays in seeking debt advice. Only 7 (7.1%) of those delay regrets were for action whereas 92 (92.9%) were for inaction. On the other hand, 298 (75.3%) of all other regrets were for action whereas only 98 (24.7%) of them were for inaction. As might be expected, the association between delay regrets and inaction was statistically significant, χ^2^(1) = 152.81, *p* < 0.001.

### Associations between mental health and regret and contributory factors

Because of the ceiling effect in regret intensity ratings, we were unable to test the prediction of a negative association between regret intensity and well-being.

Zero order correlations between mental health and ratings of factors contributing to the debt are presented in Table 1. Although all of the correlations presented in the table are weak, the correlation between GHQ12 scores and ratings for the contribution made by employment issues was positive and statistically significant.

To examine associations between regret content and well-being we calculated mean GHQ12 scores for participants broken down by whether they reported or did not report at least one of the five regret types. These means and associated standard deviations are to be found in Table 3. T-tests on these means (see Table 3) revealed that only the association between GHQ12 scores and whether participants reported at least one regret for events beyond their control was statistically significant.

**Table 3:** Mean GHQ12 scores broken down by whether participants described at least one regret of each type

	Mean (SD) GHQ12				
Regret Content	No regrets	> 1 regrets	t (df)	P	d
Spending	14.81 (10.28) *N* = 108	13.29 (9.42) *N* = 143	1.22 (249)	.23	.16
Delay	14.13 (10.17) *N* = 163	13.61 (9.16) *N* = 88)	.40 (249)	.69	.05
Events	12.66 (9.10) *N* = 159	16.17 (10.62) *N* = 92	2.77 (249)	.006	.36
Consequence	13.97 (9.71) *N* = 145	13.92 (9.99) *N* = 106	.046 (249)	.96	.006
Character	13.57 (9.29) *N* = 134	14.38 (10.39) *N* = 117	.65 (249)	.52	.08
Other	13.51 (9.72) *N* = 173)	14.92 (10.00) *N* = 78	1.06 (249)	.29	.14

T-statistics test for significant associations between regret and GHQ12 scores

## Discussion

The results we have described here bear on all six of the research questions outlined at the outset. First, people resolving problem debts differ in the extent to which they perceive a variety of factors as having contributed to their debt problems. Although participants indicated that living beyond their means and bad financial decisions made the biggest contribution to their debt problems, other factors, such as employment, relationship, and health problems also played an important role. Second, people tend to express regret for spending or financial decisions significantly more than other types of regret, but such regrets were not as prevalent as might have been expected given participants’ circumstances, and both character regrets and regrets about delays in advice seeking are also prevalent. Third, the tendency to have a significantly greater proportion of one’s regrets related to spending was only observed amongst participants who gave higher ratings for spending and financial decisions as contributory factors. Strikingly, however, less than half of these participants’ regrets were of spending, and more than one third of them did not describe a regret related to spending at all.

Fourth, and contrary to the tendency to regret inaction more than action which is usually described in the literature [19, 25] we found that regrets for action were significantly more prevalent than regrets for inaction. Interestingly, this tendency was reversed amongst regrets for delays in advice seeking.

Our fifth question concerned associations between mental health and both participants’ ratings of the factors that contributed to their problem debt and the content of their regrets. Strikingly, we found significant associations between well-being and both ratings of employment issues as a contributing factor and regrets relating to issues beyond the participants’ control. Both of these findings suggest that certain pathways into indebtedness, particularly ones over which the debtor lacks control, may make them more vulnerable to mental health problems. Surprisingly, we found that participants who reported waiting longer before seeking advice also had better well-being scores. Given that waiting to seek debt advice was also associated with higher debt levels, it is possible that better mental health reflects resilience which raises the paradoxical possibility that in some cases, better mental health might cause debtors to behave in ways that indirectly lead to exacerbation of their debt problems.

Finally, we found notable gender differences. Consistent with earlier research [18], men reported having bigger debts than women. Men also rated spending beyond their means as a bigger contributing factor than did women and reported waited longer before seeking advice than did women. Goode [29] identifies a number of reasons why men wait longer than women before seeking advice, ranging from men seeing it as their role to ‘fix’ household problems, including financial ones, and shame experienced about needing to ask for help. Consistent also with recent work demonstrating that debt problems in middle- and older-aged women are associated with being divorced or separated [55], we found that women rated relationship problems as a bigger contributing factor than did men. Contrary to previous findings in the literature on financial decision making (e.g., [4, 14]), we found no evidence of differences due to gender in the content of debtors’ regrets.

## How debts become problematic

Our results strongly suggest that people may explain their journey into problem debt in different ways: while many participants attributed importance to their own financial decision-making others attributed importance to life course factors. Participants in this latter group rated employment, relationship and health issues as being important to their debt problems. Crucially, ratings for the six contributory factors were not correlated with each other to any great extent. That means that participants do not fall neatly into one group that attributed their debt problems to poor decision making and another that emphasised life course factors. This conclusion is supported by the patterns observed in the regret data. People’s regrets did pattern somewhat consistently with the importance that they attributed to different causal factors in accounting for their debt problems. For example, spending regrets were more pronounced for people who attributed their debts to overspending or poor financial decision-making. Such people were also less likely to regret events beyond their control than those who did not place such an emphasis on financial decision making in explaining their debt problems. However, even amongst participants who were at or above the median on the spending measure, the modal proportion of spending regrets was zero. This result suggests that participants were likely to have had multi-factorial accounts of their pathways to problem debt. Thus, although there is good evidence of dispositional and life course pathways into debt [69], problem debtors may not think of themselves as following one or other pathway. Ratings of contributory factors and patterns in the regret content suggest that many participants perceive both their own decisions about money and life events to have played a role in their problems.

Much of the evidence about pathways into debt comes from general population studies. Indeed, the idea of a life course pathway into debt is that such debt is taken on in the expectation of increased future income (see [18, 69]. Our data suggest that debt taken out for rational reasons has the potential to become problematic when unexpected life events, such as losing one’s job or a health issue, interfere with one’s ability to service the debt. Recent work emphasises associations between debt and more predictable events in the life course. For example, Oksanen, Aaltonen & Rantala [52] find an association between problem debt and both leaving home and, for men, having a first child. Notably, the relationship between having a first child and debt problems takes several years to emerge and, of course, not every new young father goes on to develop problem debts. The data reported here suggest that, from the point of view of the debtor, both imprudent financial decision making and uncontrollable events may play a role in determining whose debts become problematic following a more predictable life course event.

## What people in debt regret

Participants in our sample had, by definition, experienced bad financial outcomes. Surprisingly, even amongst those who considered their own financial decisions to have played a role in those outcomes, the majority of their regrets did not relate to those decisions and many of them expressed no regret for their bad financial decisions. This can be interpreted as evidence that myopic decisions tend not to be regretted, particularly with the passing of time, as frequently as might be imagined. This result is also consistent with the results of previous work on impulsive and compulsive shopping [6, 20] suggesting that regret for myopic spending is not ubiquitous, even when participants are asked to consider individual purchases. Indeed, Dittmar & Drury [20] point out that although standard economic models of choice expect regret to inevitably follow poor or impulsive decision making, there is sufficient difference between individuals as to call that expectation into question. Arguably, our finding that even amongst people who attributed their debt to financial decisions, less than 50% of regrets concerned spending, also calls that same expectation into question.

People’s real-life regrets appear to be for inaction rather than action, particularly in the long run (see [19, 25, 27, 75]). Gilovich & Medvec [25] appeal to a number of mechanisms that come into play with the passing of time when accounting for this finding including the inexplicability of inaction relative to action (see also [15]) and people’s ability to find silver linings for their regretted actions rather than for their regretted inactions. Strikingly, participants in our study were significantly more likely to express regret for action than they were for inaction. This is despite the fact that, on average, it took them over a year to seek help for their debt problems. That is, at least a year had passed since the decisions and events which they regretted. As there are multiple determinants of people’s tendency to attribute their regrets to inaction (see [19, 25]), there are likely to be a number of important factors here. One possibility is that faced with wholly negative and ongoing financial problems, debtors are unable either to justify their earlier financial decisions or to find silver linings in the outcomes of those decisions. An exception to the pattern described above is the large percentage of participants who attributed their regret for decisions to delay seeking debt advice to inaction rather than action. Often participants who expressed such regrets also wrote that they tried to address the problem themselves. The frequency with which such regrets were expressed suggests that they could be used as the basis for messaging designed to encourage people with problem debts to engage with debt advice services.

## Implications for debt advice services

The results described here have implications for those engaged in the provision of debt advice. Perhaps most obviously, and in line with other studies (see [56]), our findings suggest that debt advisors should be aware that indebted clients are more likely than non-indebted individuals to be suffering from poor mental health. Moreover, beyond their indebtedness, our results suggest that the explanation offered by clients for their indebtedness may be predictive of mental health problems. In particular, explanations which appeal to factors beyond the debtor’s control, such as loss of employment, may be associated with poorer mental health. Future work will be need to establish the basis for this association but from a practical point of view, while the provision of good debt advice likely requires the advisor to be familiar with the client’s current financial position, our results suggest that knowledge of how the client perceives themselves to have arrived at that position may also be important. Gender also appears to be an important moderating factor here. For example, men report larger debts than women and women rate the contribution made by relationship problems to their problem debts significantly higher than do men. The latter finding is consistent with earlier work on financial problems amongst older women [55] and coheres with recent insights in the emerging literature on economic abuse (see [32]). Given our findings and those around economic abuse, there may be particular challenges posed in providing advice to women (and men) whose relationship difficulties contributed to their debt problems.

Finally, debt advisors might find it useful to be aware of the surprising direction of the link we observed between better mental health and longer delays before seeking debt advice such that longer delays were associated with better well-being. Although the cause of this relationship is unclear, one interpretation is that more resilient debtors initially attempt to resolve their debts on their own, without seeking advice. We do not know the triggers for seeking debt advice in this more resilient group, but apart from the delay possibly exacerbating their debt problems, there may be different challenges posed to debt advisors by the provision of advice to groups with better versus poorer mental health. Future research might address this question.

## Limitations and future work

Although we wish to draw general conclusions about debtors’ regrets, we studied predominantly one type of problem debtor, and all of our participants were clients of a single debt resolution company. IVAs are intended for debtors who meet a particular set of criteria and hence it is possible that our results would have been different had a wider range of problem debtors been included. Our data were collected in the aftermath of the financial crisis of 2007–8. As macro-economic causes and micro-economic consequences are likely to differ between financial crises, our findings (particularly those relating to regret content) may contain results that are idiosyncratic to the time at which the data were collected. Although we think it likely that many of the findings from our study are likely to be replicated during other financial crises, more work will be required to establish their generality.

Future work might also directly test our suggestion that we observed a large proportion of debtors with no financial or spending regrets because ‘hot’ regrets about myopic financial decisions decreased over time. An alternative possibility is that people who end up with problem debts experience different emotional responses to bad financial outcomes in the immediate aftermath of those outcomes becoming known. One way to begin to test this alternative hypothesis in future work would be to use experimental tasks (see [16, 46]) to compare the financial decisions and emotional responses of people engaging with problem debts to controls.

There is also scope for more detailed study of debtors’ beliefs about their individual regrets. For example, accounts of what people regret differ in terms of the importance they ascribe to future opportunities to make similar choices [7], [58]. It would have been interesting to assess relations between participants’ beliefs about their financial futures and whether they believed that financial rehabilitation was possible for them. Moreover, future studies might measure participants’ financial self-efficacy [44] and its links both to regret and time taken to seek debt advice. An individual’s financial self-efficacy is the extent to which they have a sense of agency about financial decisions and low financial self-efficacy is known to be related to a higher number of debt-related financial products held [21]. Moreover, less financially vulnerable individuals tend to have higher financial self-efficacy [31]. To the best of our knowledge, the construct has not been examined in a sample with problem debts.

Finally, we have studied people in the midst of an extremely negative life event, and we believe that aspects of our data might be used to make such extreme circumstances less likely for future debtors. In particular, future work might test the efficacy of messaging based on both regret about delays to seek advice and statistical associations between delay and debt size. To the extent that such messages might help people anticipate the regret that they would feel should they not seek advice as soon as they realise they need it, they might help debtors to decide to approach debt advice agencies. Certainly, the time that participants in this study reported waiting before seeking advice suggests that such nudges are necessary.

## Conclusions

In a very hard to reach sample, we have shown that people resolving problem debts attribute their debt problems to their own financial decision making but also to life events outside their control. This pattern is consistent with existing accounts of pathways into debt. People’s regrets about their pathway into debt are more surprising; although the largest category of regrets concerned spending, even people who attributed their problems to their own financial decision making described fewer regrets of spending than of other types. It may be that the ‘hot’ regrets associated with myopic financial decisions or lack of financial discipline fade quite quickly whereas regrets about other aspects of the problem debt experience linger and maybe even increase in intensity over time. Importantly, the tendency to experience regret over uncontrollable events was associated with poorer mental health, and regrets over delays in seeking advice were common. Although educating people to avoid problem debt is very difficult (see [22]), and perhaps impossible when the debt is due to uncontrollable events, encouraging debtors to anticipate the regret they will feel should they delay in seeking advice has potential to result in more timely engagement with debt advisors.

## Supplementary Information

Supplementary Material 1.

## Data Availability

Research data are not shared due to ethical restrictions. The research was funded by the company whose clients participated in the study. Because of the sensitivity of the data collected and the potential for conflict of interest, the ethics committee that reviewed the research stipulated that only the researchers directly involved with the study should have access to the data. Participants gave their consent on the basis of information to that effect, and we cannot share the data as a consequence. We have communicated with the current ethics committee at QUB and, given changes to expectations around data sharing that have occurred in the time since we collected the data, they have given us permission to share data with an action editor and reviewers should they request to see it. However, we cannot make the data publicly available.
