# Supplementary material for: Debt-related regret and well-being in people resolving problem debts
Source: BMC Psychol. 2025 Jul 1;13:718. doi: 10.1186/s40359-025-03045-9 (PMC12220135; doi:10.1186/s40359-025-03045-9)
Supplement: Supplementary file 1 — Supplementary Material 1. [file 40359_2025_3045_MOESM1_ESM.docx]

**Supplementary Materials – Survey Items**

***Part 1: You and your background***

For questions in this section which offer a choice of answers, simply delete the responses that don’t apply to you.

1. Please indicate your age: ___

2. Are you:

A. Male

B. Female

3. What is your marital status?

A. Single

B. Married

C. Separated/Divorced

D. Other

4. Do you have children?

A. Yes

B. No

5. To what level are you educated?

A. GCSE

B. A Level

C. University

D. Other

6. Which of the following best describes your situation:

A. Filed for bankruptcy

B. Filed for Individual Voluntary Arrangement (IVA)

C. Filed for Debt Management Plan

D. Yet to decide

7. By approximately how much are you in debt?

A. Up to £20,000

B. Between £20,001 and £40,000

C. Between £40,001 - £60,000

D. Between £60,001 - £80,000

E. Between £80,001 - £100,000

F. Over £100,000

8. How many months was it after you realised that you had a serious debt problem before you sought help? ___

***Part 3: How you feel about your situation***

To what extent do you feel that the following factors contributed to your debt issues? For each issue, place an X in one of the boxes from 1 (Little/no contribution) to 5 (Large contribution).

|  | **1 – Very little/No contribution** | **2** | **3** | **4** | **5 – Very large contribution** |
| --- | --- | --- | --- | --- | --- |
| **Employment issues e.g. redundancy, unemployment** |  |  |  |  |  |
| **Relationship issues e.g. Separation or divorce, bereavement** |  |  |  |  |  |
| **Poor financial decisions e.g. bad investments, taking out a large mortgage** |  |  |  |  |  |
| **Living beyond means e.g. expensive purchases** |  |  |  |  |  |
| **Failure of a business** |  |  |  |  |  |
| **Health problems e.g. medical bills** |  |  |  |  |  |

Are there things about the run-up to your financial difficulties that you regret? If so, please describe below up to three things about which you feel regret:

Regret A:

Please indicate how much regret you feel when you think about what you have described above by entering a number from 1 to 5, where 1 means "very little regret" and 5 means "a lot of regret". Delete the numbers until your choice remains:

Very little regret 1 - 2 - 3 - 4 - 5 A lot of regret

Regret B:

Please indicate how much regret you feel when you think about what you have described above by entering a number from 1 to 5, where 1 means "very little regret" and 5 means "a lot of regret". Delete the numbers until your choice remains:

Very little regret 1 - 2 - 3 - 4 - 5 A lot of regret

Regret C:

Please indicate how much regret you feel when you think about what you have described above by entering a number from 1 to 5, where 1 means "very little regret" and 5 means "a lot of regret". Delete the numbers until your choice remains:

Very little regret 1 - 2 - 3 - 4 - 5 A lot of regret

***Part 4: Your current well-being***

The following 12 questions concern your general psychological well-being recently. Each question has four responses. For each statement, delete the responses that don’t apply to you, until your choice remains.

Have you recently:

1. been able to concentrate on what you’re doing?

A. Better than usual B. Same as usual C. Less than usual

D. Much less than usual

2. lost much sleep over worry?

A. Not at all B. No more than usual C. Rather more than usual.

D. Much more than usual

3. felt that your are playing a useful part in things?

A. More so than usual B. Same as usual C. Less so than usual

D. Much less so than usual

4. felt capable of making decisions about things?

A. More so than usual B. Same as usual C. Less so than usual

D. Much less than usual

5. felt constantly under strain?

A. Not at all B. More than usual C. Rather more than usual

D. Much more than usual

6. felt you couldn’t overcome your difficulties?

A. Not at all B. No more than usual C. Rather more than usual

D. Much more than usual

7. been able to enjoy your normal day-to-day activities?

A. More so than usual B. Same as usual C. Less so than usual

D. Much more than usual

8. been able to face up to your problems?

A. More so than usual B. Same as usual C. Less so than usual

D. Much less than usual

9. been feeling unhappy and depressed?

A. Not at all B. No more than usual C. Rather more than usual

D. Much more than usual

10. been losing confidence in yourself?

A. Not at all B. No more than usual C. Rather more than usual

D. Much more than usual

11. been thinking of yourself as a worthless person?

A. Not at all B. No more than usual C. Rather more than usual

D. Much more than usual

12. been feeling reasonably happy; all things considered?

A. More so than usual B. Same as usual C. Less so than usual

D. Much less than usual
